# Patient Satisfaction and Recommendations for Delivering a Group-Based Intensive Outpatient Program via Telemental Health During the COVID-19 Pandemic: Cross-sectional Cohort Study

**DOI:** 10.2196/30204

**Published:** 2022-01-28

**Authors:** Michelle K Skime, Ajeng J Puspitasari, Melanie T Gentry, Dagoberto Heredia Jr, Craig N Sawchuk, Wendy R Moore, Monica J Taylor-Desir, Kathryn M Schak

**Affiliations:** 1 Department of Psychiatry and Psychology Mayo Clinic Rochester, MN United States; 2 Department of Nursing Mayo Clinic Rochester, MN United States

**Keywords:** COVID-19, telemental health, teletherapy, telepsychiatry, telemedicine, intensive outpatient, patient satisfaction

## Abstract

**Background:**

Although group-based intensive outpatient programs (IOPs) are a level of care commonly utilized by adults with serious mental illness, few studies have examined the acceptability of group-based IOPs that required rapid transition to a telemental health (TMH) format during the COVID-19 pandemic.

**Objective:**

The aim of this study was to evaluate patient satisfaction and future recommendations for a group-based IOP that was transitioned to a TMH format during the COVID-19 pandemic.

**Methods:**

A 17-item patient satisfaction questionnaire was completed by patients at discharge and covered 3 areas: IOP TMH satisfaction, future recommendations, and video technology challenges. Descriptive and content analyses were conducted for the quantitative and open-ended questions, respectively.

**Results:**

A total of 76 patients completed the program in 2020. A subset of patients (n=40, 53%) responded to the survey at program discharge. The results indicated that the patients were satisfied overall with the TMH program format; 50% (n=20) of the patients preferred the program continue offering the TMH format, and the rest preferred returning to in-person formats after the pandemic. The patients indicated the elements of the program that they found most valuable and provided recommendations for future program improvement.

**Conclusions:**

Overall, adults with serious mental illness reported high satisfaction with the group-based IOP delivered via TMH. Health care systems may want to consider offering both TMH and in-person formats regardless of the state of the pandemic. Patients’ feedback on future improvements should be considered to help ensure long-term success.

## Introduction

The COVID-19 pandemic has led to increased demand for mental health services worldwide, and most countries are reporting significant disruptions to the delivery of critical mental health services [1]. Early evidence suggests that symptoms of anxiety, depression, and self-reported stress were common responses to COVID-19 in the general population [2]. Concerns that suicide rates during and after the pandemic might increase have been highlighted [3], though data are still limited on the rates and risk of suicide in the context of the current pandemic. Certain populations, such as those with serious mental illness (SMI), may be particularly vulnerable to the stressors and hardships related to COVID-19. Thus, it is pertinent to ensure adequate access to behavioral health services during this pandemic, particularly for adults with SMI.

The COVID-19 pandemic has created significant obstacles to the delivery of mental health services, especially for services delivered in a group setting due to the need for social distancing. However, maintaining access to group-based interventions is essential given their efficiency in treatment delivery to a larger population when resources are limited. Telemental health (TMH), defined as the delivery of mental health care services at a distance through the use of information and telecommunications technology, has emerged during the COVID-19 pandemic as an essential platform to ensure continuous mental health care delivery. TMH has been shown to be highly effective and increases access to care [4]. It has been shown to be an effective mode of health care delivery across different patient populations, diagnoses, and settings, including group interventions [5-7]. The COVID-19 Federal Emergency Order temporarily lifted several administrative barriers to TMH, allowing for its expanded use during the pandemic [8]. As a result, TMH services have been increasing substantially in the wake of COVID-19, with the veterans administration reporting a 500% increase in TMH use in the early stages of the pandemic [9]. Initial TMH studies during the pandemic have shown increased utilization and decreased no-show rates [10]. Though TMH has provided essential mental health care during this time, questions remain regarding how different populations accept and respond to TMH interventions. A study of patient satisfaction related to TMH services during the perinatal period showed that a majority of participants indicated that TMH improved their health care access and that the visit was as effective as in-person visits [11]. Understanding patient satisfaction and engagement with TMH interventions is crucial to the sustainability of TMH programs both during and beyond the pandemic.

Understanding patients’ perspective on the quality of behavioral health services delivered via telehealth is important to ensure their engagement with treatment and to improve outcomes. Several pre–COVID-19 studies indicated that patients had a positive perception toward telehealth and were satisfied with the delivery format [12]. Although the literature is still limited, studies are also finding high patient satisfaction with telehealth programs developed during the pandemic [13,14]. Emerging research during this pandemic were consistent with previous findings indicating that patients were satisfied with the option to continue behavioral health services via telehealth. Most of this research, however, has focused on individual outpatient behavioral health services. A gap in the literature exists on patient satisfaction for group-based intensive outpatient programs (IOP) delivered via telehealth during the pandemic.

The aim of this study was to evaluate patient satisfaction while exploring future recommendations of a group-based IOP for adults with SMI, which was rapidly transformed to a telehealth format during the COVID-19 pandemic. The results from this study can be used to improve the quality of programming and enhance the delivery of services in the future.

## Methods

The protocol for this cross-sectional cohort survey research was approved by the Mayo Clinic Institutional Review Board. Data were collected as part of clinical care at the Adult Transitions Program (ATP), a group-based IOP within the Mayo Clinic Department of Psychiatry and Psychology. This program was intended to treat adults with SMI who were recently discharged from psychiatric hospitalization or were at risk of psychiatric hospitalization if not treated in a more intensive level of outpatient care. Inclusion criteria for the present study were patients who were admitted to ATP, were at least 18 years old, and consented for their clinical data to be used for research purposes. The patients completed the satisfaction survey over the phone with research personnel after they were discharged from the program. The phone call took approximately 15 minutes to complete.

ATP was delivered by a multidisciplinary team that included psychologists, a psychiatrist, nurse practitioners or physician assistants, licensed professional clinical counselors, occupational therapists, and registered nurses. The patients received the program 5 days per week, 3 hours a day, for a 3-week period. The programming was mainly group-based and informed by evidence-based cognitive and behavioral interventions such as Behavioral Activation [15], dialectical behavioral therapy (DBT) [16], and acceptance and commitment therapy [17]. The patients were assigned to 1 of the 3 tracks, with 8 patients in each track. The inclusion criteria for the program were adults aged 18 years and older, who were diagnosed with SMI (eg, mood disorders, anxiety disorders, psychosis, personality disorders, and substance use), who had recent psychiatric hospitalization or were at risk for psychiatric hospitalization, and who reported having access to a mobile or computer device to connect to the video teleconference software (ie, Zoom). The exclusion criteria were cognitive impairment and higher symptom severity that did not require a higher level of care with a psychiatric hospitalization or residential settings.

The patient satisfaction questionnaire was developed through a literature review. Some items were generated based on the acceptability of intervention measure, intervention appropriateness measure, and feasibility of intervention measure by Weiner and colleagues [18]. These original measures have Cronbach alphas from .85 to .91, and test-retest reliability coefficients ranged from 0.73 to 0.88. The research team generated and reviewed the initial items, and the suggested changes included adding and removing certain questions and improving grammatical errors and wording. The research team members took each iteration of the survey to ensure the readability of the content items. The final version of the Patient Satisfaction Questionnaire (Multimedia Appendix 1) included 14 quantitative questions answered on a Likert-type scale from 1 to 5 with the higher numbers indicating higher satisfaction. Three open-ended questions assessed the patients’ overall experience with TMH, the most valuable part of the TMH format, and recommendations for future program improvement. In addition, demographic variables were pulled from the electronic health record.

Descriptive statistics were generated to identify the most commonly endorsed items. The open-ended questions were analyzed using summative content analysis [19]. Keywords were identified and quantified to characterize the themes that emerged from the 3 open-ended questions. Two researchers independently read the qualitative responses multiple times to identify the keywords. These keywords were then sorted into categories, and the themes were then quantified using frequency counts. The 2 researchers compared emerging categories for validation purposes.

## Results

A total of 76 patients were admitted to the program between March and August of 2020. Of the 76 patients admitted to the program, 40 (53%) completed the survey over the phone with research personnel. The referral source and track attended for those who did and did not complete the survey were similar. The referral source for completers versus noncompleters, respectively, was as follows: inpatient, 42.5% versus 35%; emergency department, 2.5% for both groups; primary care, 30% versus 27.5%; other outpatient programs, 15% versus 32.5%; and other programs, 10% versus 2.5%. The track attended for the completers versus noncompleters, respectively, were as follows: cognitive behavioral therapy morning 25% versus 30%; DBT morning 30% versus 42.5%; and DBT afternoon 45% versus 27.5%. The patients had a mean age of 36.55 (SD 13.43) years. The majority of the patients were female (n=32, 80%) and White (n=33, 82.5%), married (n=14, 35%) or single (n=23, 57.5%), cisgender (n=38, 95%), heterosexual (n=30, 75%), and employed (n=23, 57.5%). The patients had the following psychiatric diagnoses as a primary presenting problem: major depressive disorder (n=29, 72.5%), anxiety disorder (n=2, 5%), borderline personality disorder (n=6, 15%), and suicidal ideation (n=2, 5%). Full baseline characteristics are reported in Table 1.

**Table 1:** Baseline characteristics of study sample.

Characteristics			Values
**Gender, n (%)**			
	Female	32 (80)	
	Male	6 (15)	
	Transgender female or male to female	1 (2.5)	
	Nonbinary or genderqueer	1 (2.5)	
Age (years), mean (SD)			36.55 (13.43)
**Race, n (%)**			
	White	33 (82.5)	
	Other	6 (15)	
	African American	1 (2.5)	
**Ethnicity, n (%)**			
	Hispanic or Latino	3 (7.5)	
	Non-Hispanic or Latino	36 (90)	
	Unknown	1 (2.5)	
**Marital status, n (%)**			
	Single	23 (57.5)	
	Married	14 (35)	
	Separated	2 (5)	
	Divorced	1 (2.5)	
**Employment, n (%)**			
	Currently employed	23 (57.5)	
	Not employed	14 (35)	
	Disabled	3 (7.5)	
**Financial resource strain, n (%)**			
	Not hard at all	17 (42.5)	
	Not very hard	8 (20)	
	Somewhat hard	10 (25)	
	Hard	2 (5)	
	Very hard	1 (2.5)	
	Not on file	2 (5)	
**Sexual orientation, n (%)**			
	Lesbian or gay	2 (5)	
	Heterosexual	30 (75)	
	Something else	1 (2.5)	
	Don’t know	2 (5)	
	Choose not to disclose	1 (2.5)	
**Presenting problems, n (%)**			
	Major depressive disorder	29 (72.5)	
	Suicidal ideation	2 (5)	
	Anxiety disorder	2 (5)	
	Borderline personality disorder	6 (15)	
	Other	1 (2.5)	
**Comorbidity, n (%)**			
	Yes	17 (42.5)	
	No	23 (57.5)	
**Track, n (%)**			
	DBT^a^ morning	12 (30)	
	DBT afternoon	18 (45)	
	CBT^b^ morning	10 (25)	
**Source of referral, n (%)**			
	Inpatient	17 (42.5)	
	Emergency department	1 (2.5)	
	Primary care	12 (30)	
	Other outpatient	6 (15)	
	Other programs	4 (10)	
Days completed, mean (SD)			14.4 (1.5)
Program absences (days), mean (SD)			0.7 (1.6)
**Program absences (days), n (%)**			
	None	28 (70)	
	1-3	10 (25)	
	4-7	2 (5)	

^a^DBT: dialectical behavioral therapy.

^b^CBT: cognitive behavioral therapy.

The complete results for the quantitative portion of the satisfaction survey are presented in Table 2. Overall, the majority of patients reported high satisfaction, comfort, appropriateness, relevance, and compatibility of the TMH format of ATP. Most patients (92.5% [n=37]) reported that they would recommend this service format to a friend or family member. They noted that the TMH format was well organized and executed, user friendly, and not burdensome. We also assessed preference between in-person versus a TMH format. We found a split among the patients where 35% (n=14) preferred to receive an in-person format, 50% (n=20) preferred continuing with a TMH format, and 15% (n=6) were neutral when asked, “Once COVID-19 travel restrictions are lifted, would you still want to continue with video format?” (Table 2).

**Table 2:** Satisfaction survey results.

Survey items			(1), n (%)	(2), n (%)	(3), n (%)	(4), n (%)	(5), n (%)
How did the care you received over video compare to a regular in-person health care visit?			2 (5)	2 (5)	11 (27.5)	12 (30)	13 (32.5)
How willing are you to use the video visit system in the near future?			1 (2.5)	1 (2.5)	4 (10)	5 (12.50)	29 (72.5)
Would you recommend this service to a friend or family member?			1 (2.5)	0 (0)	2 (5)	5 (12.5)	32 (80)
If you could choose between receiving the service in person versus video visit, which would you prefer?			12 (30)	1 (2.5)	10 (25)	5 (12.5)	12 (30)
To what extent are you satisfied with the video format of the service that you received?			1 (2.5)	2 (5)	2 (5)	15 (37.5)	20 (50)
How well-organized and well-executed was the video format of the service that you received?			1 (2.5)	0 (0)	1 (2.5)	13 (32.5)	25 (62.5)
How comfortable are you with the video format of the service that you received?			1 (2.5)	1 (2.5)	3 (7.5)	12 (30)	23 (57.5)
How user-friendly is the video format of the service that you received?			1 (2.5)	0 (0)	4 (10)	14 (35)	21 (52.5)
How burdensome it is to receive the service via video?^a^			1 (2.5)	2 (5)	3 (7.5)	9 (22.5)	25 (62.5)
How compatible was the video visit with access to devices (eg, cell phone and computer) that you already have?			1 (2.5)	0 (0)	4 (10)	8 (20)	27 (67.5)
How appropriate is it to receive the service via video versus in person?			0 (0)	0 (0)	8 (20)	11 (27.5)	21 (52.5)
How relevant is it to receive the video format versus the in-person format in your current life context?			0 (0)	1 (2.5)	4 (10)	2 (5)	33 (82.5)
Once COVID-19 travel restrictions are lifted, would you still want to continue with video format?			10 (25)	4 (10)	6 (15)	6 (15)	14 (35)
**Did you have any difficulty with the telemental health format and video technology?**							
	Yes	18 (46.15)					
	No					21 (53.85)	

^a^Reversed item.

We also assessed to what extent patients experienced technological difficulties with the TMH format. A portion of the patients (46.15% [n=18]) reported experiencing challenges during the program. We analyzed the qualitative open-ended responses and reported that challenges included problems with slow internet connection, the video camera of their devices, logging into the teleconference room, and being inadvertently removed from the session.

We conducted content analyses of the qualitative questions and extracted themes from each question. The frequency counts for the categories within each question are presented in Table 3. Examples of the qualitative feedback are presented in Table 4.

**Table 3:** Qualitative feedback.

Questions and categories		Values, n (%)
**Patients’ perceptions of the TMH^a^ format**		
	Positive attitudes toward the format and program	28 (70)
	Increased access to treatment	6 (15)
	Treatment was effective and beneficial	8 (20)
	Increased social support	4 (10)
	Preferred in-person format	7 (18)
	Technological issues	8 (20)
	Negative attitudes towards the format and program	2 (5)
**Most valuable part of the TMH format and the program**		
	Social support	9 (23)
	Learning coping skills	5 (13)
	The convenience that telemedicine offers	27 (68)
	No valuable experience	1 (3)
**Recommendations for future improvement**		
	Improvement on the technology or TMH delivery process	5 (13)
	Improvement on therapy materials	3 (8)
	Improvement on therapeutic process or delivery	5 (13)
	Offering in-person format	1 (3)
	No further recommendations	25 (63)

^a^TMH: telemental health.

**Table 4:** Examples of qualitative responses.

Questions and categories		Sample responses
**Patients’ perception of the TMH^a^ format**		
	Positive attitudes toward the format and program	“I thought it was nice… I don’t mind the telehealth format. It was a lot organized. Each group was timed very well. I thought it was very pleasant for the most part” “I was really happy with it. In fact I still use telehealth to communicate with my other providers. This is really good. I am really thankful and grateful for it.”
	Increased access to treatment	“I am glad I had the option to continue receiving treatment via telehealth during COVID” “I think it was really good especially because I live in Michigan so it would be challenging to find a different program.”
	Treatment was effective and beneficial	“I thought it was weird starting off but actually it was still just like being in a room full of people. Honestly, I think it saved my life.” “So that is the positive of video format to use the skills immediately in my home environment.”
	Increased social support	“it was good to see other people over video” “It’s nice to see everyone while still feeling safe.”
	Preferred in-person format	“For me it is easier to do it in person. I think I would get more out of the program if it is in person.” “I very much prefer face to face. It felt more welcoming. With video you can only answer the questions. there couldn’t really be a discussion like if we have face to face and sitting in the same room.”
	Technological issues	“It was just hard to log on sometimes.” “A few times I was disconnected but that could have been on my end”
	Negative attitudes towards the format and program	“I didn’t like it. I don’t like video format.”
**Most valuable part of the TMH format and the program**		
	Social support	“Being able to still see other patients in group via Zoom.” “You get to interact with everyone still just like when you are in person.”
	Learning coping skills	“It gave me tools to overcome depression and anxiety. It gave you the tools, it just you have to learn and use it.” “You learned so much. It’s not like information overload. I’m someone who learns that way. The coping skills and being able to be honest were phenomenal.”
	The convenience that TMH offers	“The flexibility that we could do it from anywhere.” “Just being able to continue receiving therapy and not being cut off because of COVID. It is good to have it as an option.”
	No valuable experience	“I didn’t really value the program because it was in the video format.”
**Recommendations for future improvement**		
	Improvement on the technology or TMH delivery process	“Using more of the Zoom features such as the whiteboard.” “There are ways where you could have people type on the screen, I would actually use that feature more on Zoom.”
	Improvement on therapy materials	“I found a few easy things that will make the binder easier, maybe some tabs to find things [easier]” “Maybe just making sure that we get the binder and number the pages. Or maybe give the blank copy of the materials. Maybe improving the structure of the binder. And maybe to be able to send the powerpoint and all the learning tools.”
	Improvement on therapeutic process or delivery	“Maybe allow for more collaboration among the patients. They did that though in DBT group but maybe a bit more.” “The provider should be organized and know what they are teaching and explaining. Other than that they didn’t see any real issue.”
	Offering in-person format	“I do wish it could be in person.”
	No further recommendations	“No, I like everything about the video format.” “No. I don’t think so.”

^a^TMH: telemental health.

Regarding the patients’ overall perception of the TMH program, they provided both positive feedback and challenges that they encountered. The patients provided overall positive attitudes toward the TMH format. They noted that TMH provided easier access to treatment and that treatment was effective and beneficial to learning skills and coping with their problems. Some individuals also reported that TMH increased social support during the pandemic. These findings are similar to those found by Ackerman et al [11], which showed increased satisfaction with TMH. Others noted challenges of this delivery format, which included experiencing technological issues, with one patient reporting an overall negative experience with the program. Some patients (18% [n=7]) also expressed preferences to receive services in-person rather than via TMH.

We asked the patients to identify the most valuable part of the program. More than half of the patients stated that they found the convenience of TMH as valuable, with others reporting the benefits from social support and the adequate learning skills to cope with their presenting problems.

Most patients did not provide further recommendations to improve the TMH program format. Some suggested improvements on the TMH delivery process, such as using more features on Zoom. Others suggested that the therapeutic delivery process and materials could be improved. One patient suggested that we offer the in-person format again once the pandemic is over.

## Discussion

### Principal Findings

Prior to the COVID-19 pandemic, very little information existed in the empirical literature on how to rapidly convert group-based IOPs to a TMH format. This study assessed the acceptability of a group-based IOP delivered via TMH during the COVID-19 pandemic. Our data show that patients were satisfied with the TMH ATP, and IOP, with most reporting that they would recommend these services to a friend or family member. When asked to describe their preference, most patients preferred to continue the TMH format during the pandemic and beyond. These results demonstrate that a “hybrid” model of care, which allows for both approaches (depending upon the patient’s choice and availability of stable internet services in their area) may be a viable alternative. Common technological difficulties experienced by patients included slow or unstable internet connections, malfunctioning cameras, and log-in difficulties. However, for most patients, these technological difficulties did not negatively affect their experience with the program. TMH services are important in reaching patients that are geographically distanced from mental health facilities. It is important to recognize that the infrastructure for stable internet connections within communities and access to devices that can facilitate this type of treatment play a role in who can access TMH.

Content analyses of qualitative data suggest that the patients were willing to effectively address technological problems in the spirit of accessing convenient, in-home services that reduce the risk of health care-associated infections during the COVID-19 pandemic. Further, patients noted that the TMH format facilitated the acquisition of evidence-based coping skills and engendered a sense of social connection despite ongoing social and physical distancing measures. These findings suggest that TMH IOPs are sustainable and acceptable to adults with SMI. Moreover, mental health systems should consider offering both TMH and traditional in-person services to best meet the needs of patients with diverse preferences, technologic capabilities, and learning needs regardless of the state of the pandemic.

The lack of patient-identified quality improvement recommendations is likely due to the high degree of satisfaction reported by the overall sample. Start-point recommendations offered by respondents included expanding platform features (eg, using the virtual whiteboard), improving the use of program handouts (eg, sending documents virtually) and maintaining the availability of in-person IOPs for those who prefer face-to-face treatment.

### Limitations

This study used the data gathered through convenience sampling, which limits the generalizability of our findings to other populations. Although TMH IOPs may be helpful for a large proportion of adults with SMI, not all clinics or programs may be prepared to provide such services. This study was performed at a large clinical and academic center with previous experience with telehealth programming. There was also significant administrative and information technology support available, which limits the generalizability of our findings to other clinics. Additionally, to determine patient satisfaction, we used selected items from established measures of acceptability of interventions, which may have influenced internal consistency. Furthermore, the findings may contain positive bias given that not all patients completed the satisfaction survey. Lastly, our sample lacked a comparison, in-person group, and was limited in terms of racial and ethnic diversity. This sample was also limited to those patients who had sufficient technologic knowledge, skills, and resources (eg, high-speed internet, smartphone, and computer) to engage in the TMH platform. Subsequent research should aim to report TMH IOP outcome data, ideally across a broader range of patient characteristics. Despite these limitations, the findings detailed here reinforce the benefits of delivering TMH IOPs during public health emergencies and contribute to the sparse literature available on real-world program adaptations.

### Conclusions

The COVID-19 pandemic led to the rapid adoption of TMH services across mental health systems. Our findings indicate that TMH IOPs are feasible and can be an effective, safe, and convenient treatment framework for adults with SMI. High satisfaction with TMH IOP delivery and content can be achieved without compromising ongoing social and physical distancing measures. Additional research is needed to assess the efficacy of TMH IOPs in treating mental health concerns.

## Appendix

Multimedia Appendix 1 Satisfaction survey.

## Abbreviations

ATP: adult transitions program
DBT: dialectical behavioral therapy
IOP: intensive outpatient program
SMI: serious mental illness
TMH: telemental health
