# Supplementary material for: Patient Satisfaction and Recommendations for Delivering a Group-Based Intensive Outpatient Program via Telemental Health During the COVID-19 Pandemic: Cross-sectional Cohort Study
Source: JMIR Ment Health. 2022 Jan 28;9(1):e30204. doi: 10.2196/30204 (PMC8797152; doi:10.2196/30204)
Supplement: Multimedia Appendix 1 [file mental_v9i1e30204_app1.docx]

Patient Satisfaction Questionnaire

| Question | Outcomes | | | | | |
| --- | --- | --- | --- | --- | --- | --- |
| How did the care you received over video compare to a regular in person health care visit? | 1. Video visit was much worse |  | 1. No different | |  | 1. Video visit much better |
| How willing are you to use the video visit system in the near future? | 1. Will not use |  | 1. Unsure | |  | 1. Definitely will use |
| Would you recommend this service to a friend or family member? | 1. Would not recommend |  | 1. Unsure | |  | 1. Definitely will recommend |
| If you could choose between receiving the service in person versus video visit, which would you prefer? | 1. Prefer in person visit |  | 1. Either is fine | |  | 1. Prefer video visit |
| To what extent are you satisfied with the video format of the service that you received? | 1. Not at all |  | 1. Moderately | |  | 1. Extremely |
| How well organized and executed the video format of the service that you received? | 1. Not at all |  | 1. Moderately | |  | 1. Extremely |
| How comfortable are you with the video format of the service that you received? | 1. Not at all |  | 1. Moderately | |  | 1. Extremely |
| How user friendly is the video format of the service that you received? | 1. Not at all |  | 1. Moderately | |  | 1. Extremely |
| How burdensome it is to receive the service via video?^a^ | 1. Not at all |  | 1. Moderately | |  | 1. Extremely |
| How compatible was the video visit with access to devices (e.g., cell phone, computer) that you already have? | 1. Not at all |  | 1. Moderately | |  | 1. Extremely |
| How appropriate is it to receive the service via video versus in-person? | 1. Not at all |  | 1. Moderately | |  | 1. Extremely |
| How relevant is it to receive the video format versus the in-person format in your current life context? | 1. Not at all |  | 1. Moderately | |  | 1. Extremely |
| Once COVID-19 travel restrictions are lifted, would you still want to continue with video format? | 1. Not at all |  | 1. Moderately | |  | 1. Extremely |
| Please describe your experience with the video technology | Open ended question | | | | | |
| What would you consider was the most valuable part of the video format? | Open ended question | | | | | |
| Did you have any difficulty with the telemental health format and video technology? | Yes-Explain | | | No-Explain | | |
| Do you have recommendations to improve the video format? | Open ended question | | | | | |
